# The potential role of T2*-weighted multi-echo data image combination as an imaging marker for intraplaque hemorrhage in carotid plaque imaging

**DOI:** 10.1186/s12880-021-00652-x

**Published:** 2021-08-11

**Authors:** My Truong, Claes Håkansson, Makda HaileMichael, Jonas Svensson, Jimmy Lätt, Karin Markenroth Bloch, Roger Siemund, Isabel Gonçalves, Johan Wassélius

**Affiliations:** 1 Department of Medical Imaging and Physiology, Skåne University Hospital, Lund, Sweden; 2 Department of Clinical Sciences, Lund University, Lund, Sweden; 3 Lund University Bioimaging Centre, Lund University, Lund, Sweden; 4 Department of Clinical Sciences, Lund University, Malmö, Sweden; 5 Department of Cardiology, Skåne University Hospital, Malmö , Sweden; 6 Medical Radiation Physics, Department of Translational Medicine, Lund University , Lund, Sweden; 7 Department of Radiology, Skåne University Hospital, 221 85 Lund, Sweden

**Keywords:** MRI, Atherosclerosis, Carotid plaque, Vessel wall imaging, Intraplaque hemorrhage

## Abstract

**Background:**

Carotid atherosclerotic plaques with intraplaque hemorrhage (IPH) are associated with elevated stroke risk. IPH is predominantly imaged based on paramagnetic properties of the *upstream* hemoglobin degradation product methemoglobin. This is an explorative observational study to test the feasibility of a spoiled gradient echo based T2* weighted MRI sequence (3D MEDIC) for carotid plaque imaging, and to compare signs suggestive of the *downstream* degradation product hemosiderin on 3D MEDIC with signs of methemoglobin on a T1wBB sequence.

**Methods:**

Patients with recent TIA or stroke were selected based on the presence on non-calcified plaque components on CTA to promote an enriched prevalence of IPH in the material. Patients (n = 42) underwent 3T MRI with 3D MEDIC and 2D turbo spin echo T1w black blood (T1wBB). Images were independently evaluated by two neuroradiologists and Cohens Kappa was used for inter-reader agreement for each sequence.

**Results:**

The technical feasibility for 3D MEDIC, was 34/42 patients (81%). Non-calcified plaque components with susceptibility effect *without* simultaneous T1-shortening—a combination suggestive of hemosiderin, was seen in 13/34 of the plaques. An equally large group display elevated T1w signal *in combination with* signal loss on 3D MEDIC, a combination suggestive of both hemosiderin and methemoglobin. Cohen’s kappa for inter-reader agreement was 0.64 (CI 0.345–0.925) for 3D MEDIC and 0.94 (CI 0.81–1.00) for T1wBB.

**Conclusions:**

3D MEDIC shows signal loss, without elevated T1w signal on T1wBB, in non-calcified tissue in many plaques in this group of patients. If further studies, including histological verification, confirm that the 3D MEDIC susceptibility effect is indeed caused by hemosiderin, 3D MEDIC could aid in the detection of IPH, beyond elevation of T1w signal.

## Introduction

Stroke is the leading cause of adult disability and the second leading cause of death worldwide [1]. One major cause of stroke is atherosclerotic plaques in the carotid arteries [2] and the plaque composition is important in the risk assessment. MRI is widely used to assess such plaque characteristics [3–7] as intraplaque hemorrhage (IPH), thin fibrous cap and large necrotic lipid core [3, 4, 8, 9]. IPH is one of the hallmark characteristics of *vulnerable plaque*s associated with elevated stroke risk [3, 4, 10–12], also in patients with low grade stenosis [13].

Radiological diagnosis of IPH by MRI relies primarily on the paramagnetic properties of methemoglobin, which is an early degradation product from hemoglobin and the only degradation product with T1 shortening [14, 15]. Methemoglobin is therefore viewed as the major source of elevated T1w signal in tissue [14, 16, 17] and suggestive of relatively *recent* plaque hemorrhage [7, 12, 14, 15]. Identification of the downstream degradation product hemosiderin is appealing as it may be a longer lasting sign of IPH, thereby potentially adding information to the risk assessment of plaques, especially in patients with previous cerebrovascular events.

Hemosiderin has a susceptibility effect due to its super-magnetic properties and Susceptibility Weighted Imaging (SWI) [18–21] has therefore been used to identify hemosiderin or IPH in general [18, 22], but SWI is sensitive to movement artifacts and therefore not ideal for use in the neck region. To our knowledge the relationship of T1w signal and susceptibility effects in non-calcified carotid plaque components, has not been extensively studied. In clinical cervical spinal cord imaging, a spoiled gradient echo-based T2* weighted sequence called Multi-Echo Data Image Combination (MEDIC, Siemens Healthineers) has been found to be resistant to CSF flow artefacts [23] and to provide good contrast between the grey- and white matter of the spinal cord [24]. These properties could potentially also make it useful to detect hemosiderin in plaques, especially since the 3D MEDIC sequence also allows high spatial resolution.

The aim of this explorative study was to image non-calcified carotid plaque components with 3D MEDIC and compare signs of hemosiderin with elevation of T1w signal in patients with recent TIA or stroke. This population was selected to promote a high prevalence of IPH.

## Material and methods

### Patients

Initial screening for subjects was done by a neuroradiologist (MT) on the basis of CT angiographies (CTA) of the cervical and intracranial arteries between September 2014 and July 2016 at our institution. Patients were considered based on the following inclusion criteria:

1. TIA or ischemic stroke within two weeks of the examination; AND
2. An atherosclerotic carotid plaque with a non-calcified component on the symptomatic side.

Exclusion criteria were:

1. Any contraindications to MRI; OR
2. Atrial fibrillation; OR
3. Internal carotid artery occlusion on the symptomatic side; OR
4. Age bellow 18 or inability to give informed consent.

The study was approved by the Regional Ethical Review Board and written informed consent was obtained from all included patients.

### CTA protocol

Patients were examined on either of two 64-slice CT scanners (Brilliance 64 and Ingenuity, Philips, Best, The Netherlands). Intravenous iodine The CTA was performed with intravenous Iodine contrast in arterial phase, 12–15 s after administration.

Contrast medium were either 60 ml Iomeron 400 mg/ml (Bracco, Milan, Italy) or 70 ml Omnipaque 350 mg/ml (GE Healthcare, Chicago, USA), and patients were scanned from the aortic arch to the vertex, when the attenuation reached above 200 HU in the internal carotid artery at bolus pre scan.

Reconstructed and stored images: 0.8/0.4 mm (thickness/increment) source images, 3/2 mm. Maximal projection images reconstructions in axial, sagittal and coronary planes.

### Protocol parameters Philips Brilliance 64

Voltage 120 kV, reference exposure 224 mAs. Pitch 0.609, rotation time 0.5 s, collimation 64 × 0.625 mm, slice thickness 0.8 mm, increment 0.4 mm, Acquisition matrix 512 and dose modulation iDose level 3.

### Protocol parameters Philips Ingenuity

Voltage 120 kV, reference exposure 251 mAs, pitch 0.953, rotation time 0.5 s, collimation 64 × 0.625, slice thickness 0.8 mm, increment 0.4 mm, Acquisition matrix 512 and dose modulation iDose level 3.

### MRI protocol

Patients were examined on a 3 T MRI scanner (Magnetom Skyra, Siemens Healthineers, Erlangen, Germany) equipped with a dedicated eight element phased array carotid surface receive coil (Rapid Biomedical Rimpar, Germany).

The clinical protocol included pre- and post- Gadolinium-enhanced acquisitions with a total scan time of 22 min is shown in Table 1 and sequence information for the 2 sequences used in the evaluation is shown in Table 2. The real examination time for each patient however, ranged between 20 and 50 min, depending on the need for re-scans due to patient movement. The two sequences used for evaluation of intraplaque hemorrhage in this study, a fat-saturated 2D Turbo spin echo black blood sequence (T1wBB) and a 3D MEDIC sequence, were both acquired before contrast injection. Patients were in supine position with a pillow as neck support and heads slightly extended and carotid coils bilaterally positioned as close to the carotid bifurcation as possible. In cases where bilateral optimal coil positioning was difficult due to anatomic limitations such as a short neck, the symptomatic side was prioritized. Axial slices for 3D MEDIC and T1wBB were positioned perpendicular to the common carotid artery oriented after a 3D TOF with the following sequence parameters:

- T1wBB: Time of acquisition 4:26 min. 15 slices collected sequentially, slice thickness 2.0 mm, distance factor 0%, phase encoding direction A-P, FoV read 160 mm, FoV phase 75%, base resolution 320, phase resolution 100%, voxel size: 0.5 × 0.5 mm^2^ interpolated to 0.3 × 0.3 mm^2^, TR = 800 ms, TE = 10 ms, Turbo factor 11. Fat saturation strong.
- 3D MEDIC: Time of acquisition 3:54, voxel size: 0.7 × 0.7mm^2^, 60 slices, slice thickness 0,7 mm, slice oversampling 6.7%, phase encoding direction A-P, FoV 180 mm, FoV phase 100%, base resolution 256, phase resolution 100%, TR = 29 ms, TE = 16,0 ms, 3 echoes, Flip angle 8˚, Fat suppression: Water excitation normal.

**Table 1:** MRI protocol

Sequence	Time (min)
Localizer	0:13
TOF 3D	4:08
TSE 2D T1wBB	4:30
3D MEDIC	3:54
Gadolinium (Dotarem®) administration + delay*	5:0
TSE 2D T1wBB + Gd*	4:30

Estimated scan times for each sequence of the MRI protocol

^*^The post-Gd sequence was not included in this analysis, but part of the clinical MRI protocol

**Table 2:** MRI sequence parameters

Sequence parameter	TSE2D T1wBB	3D MEDIC
Acquired in-plane resolution (mm x mm)	0.5 × 0.5	0.7 × 0.7
Reconstructed in-plane resolution (mm x mm)	0.3 × 0.3	0.7 × 0.7
Slice thickness (mm)	2	0.7
Number of slices	15	60
Repetition time, TR (ms)	750	29
Echo time, TE (ms)	10	16
α°	-	8
Number of echoes	-	3
Turbo spin factor	11	-
Time duration (min: s)	4:30	3:54

MRI sequence parameters for the T1wBB and 3D MEDIC sequence

### Image analysis

All images were independently reviewed by two experienced neuroradiologists (MT and CH) (SECTRA PACS IDS7, Sectra AB, Linköping, Sweden) on dedicated high-resolution image working stations (Barco medical screens model MDCC-6430, Kortrijk, Belgium). In cases with disagreement between the observers, a consensus decision was reached, that was used for the analysis. Inter-rater agreement was calculated using Cohen’s kappa with a 95% confidence interval.

The co-localization analysis was done visually, on trans-axial CTA and MR images (3D MEDIC and T1wBB) side by side. The images were synchronized based on distinct morphologic landmarks such as macro-calcifications, plaque shape and the bifurcation. The matching of levels and synchronization of the three stacks was done by one of the reviewers and the settings were saved to ensure that both reviewers analyzed the same matched levels.

Areas within the plaque, were labelled as *non-calcified plaque components,* if the attenuation on CTA images was between 0-200HU and labelled as *calcifications* if the plaque attenuation was 500–1000 HU on CTA. Degree of stenosis on MRI and CTA was calculated by the common carotid artery method [25].

If the vessel contained more than one non-calcified plaque component, the largest was selected.

*Signal loss on 3D MEDIC* was defined as any area within a non-calcified plaque component with signal intensity lower than the ipsilateral sternocleidomastoid muscle. In cases of uncertainty, the signal in the plaque was measured within a circular ROI and compared to the measured signal value of a ROI placed in the muscle. If the signal difference was more than 1.5 times, we decided that the signal loss was significant. The classification of signal loss was binary and labeled *MEDIC* + if signal loss was seen and *MEDIC-* if signal loss was absent.

*Elevation of T1w signal* was defined as higher signal within the plaque component than in the ipsilateral sternocleidomastoid muscle and the common carotid artery on the T1wBB images.

In cases of uncertainty, the signal in the plaque was measured with a ROI tool and compared to the measured signal value found in the muscle. If the signal difference was more than 1.5 times, we decided that the elevated signal on T1wBB, was significant. The classification was binary and labelled *T1wBB* + if elevated T1w signal was seen and *T1wBB-* if absent.

## Results

### Patient population

Between September 2014 and July 2016, 1280 consecutive CTA were screened for candidates, and the 46 patients that met all inclusion criteria were considered for inclusion. Four of these had contraindications to MRI that were not known at inclusion or withdrew from participation after the initial consent.

Of the 42 patients that completed the MRI examination, 3D MEDIC images were non-diagnostic, typically due to patient motion in 8 patients, resulting in 34 patients with a full set of diagnostic images for the final image analysis (Table 3).

**Table 3:** Patient data

All patients with complete imaging (n)	34
Median age (total range, IQR)	72.5 (35–86, 66–78)
Female (%)	11 (32)
Indication	
TIA n (%)	11 (32)
Stroke n (%)	23 (68)
Degree of stenosis %	
CTA median (total range, IQR)	70 (35–95, 50–88)
MRI median (total range, IQR)	70 (35–95, 60–88)

Demographic data for the 34 patients with a full set of diagnostic images

### Image analysis

**Fig. 1:**
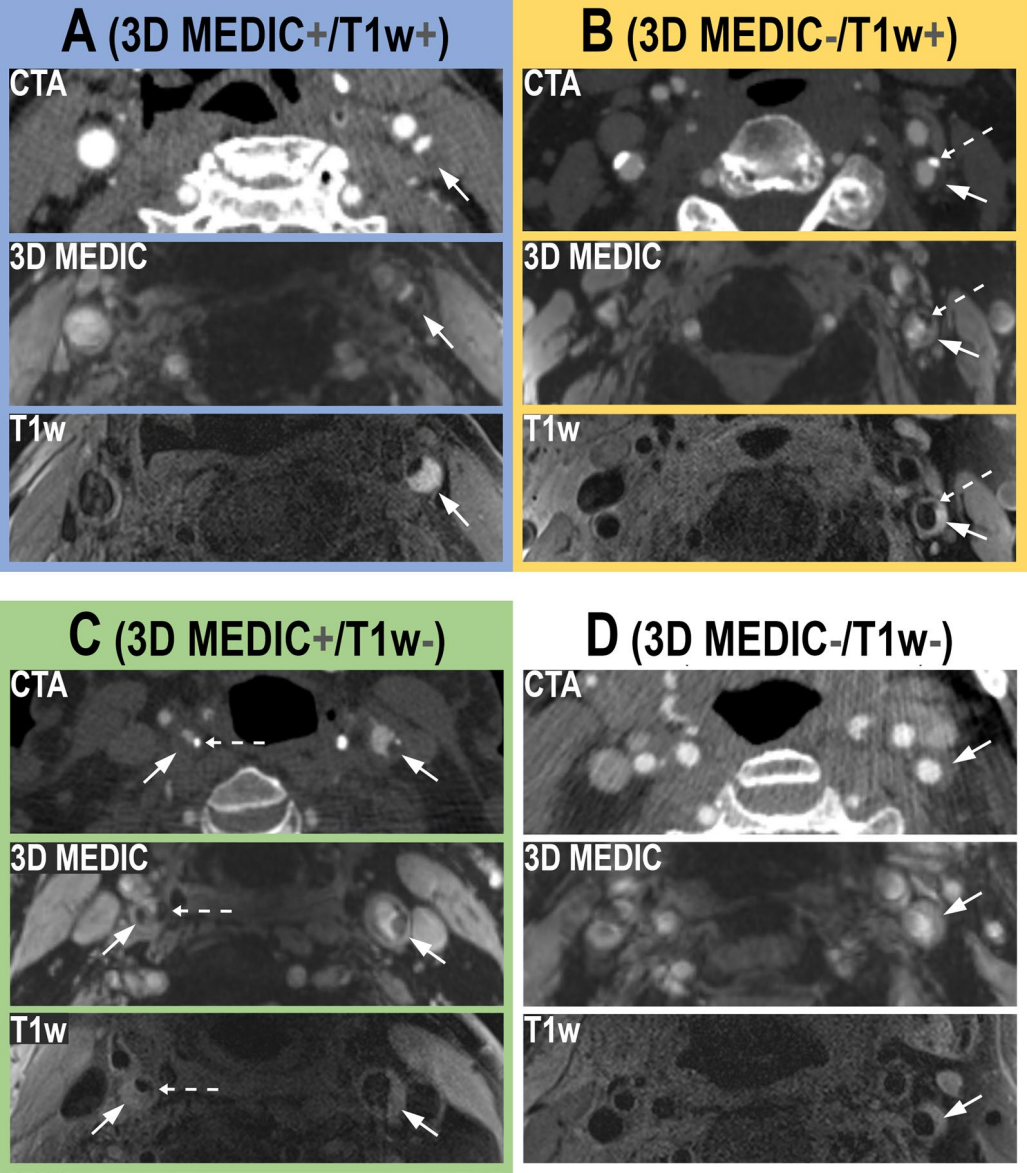
Typical imaging findings for each of the four groups at the level of the carotid bifurcation on CTA, 3D MEDIC and T1wBB, described in Table 4. The color representation for each of the four groups are the same as in Table 4. In panel **A**, in blue color, the arrow indicates a large non-calcified plaque component with signal loss on 3D MEDIC and elevated T1w signal (**3DMEDIC + /T1w +**). In panel **B**, in yellow color, the arrow indicates a non-calcified plaque component with no signal loss on 3D MEDIC and elevated T1w signal (**3DMEDIC-/T1w +**). The dotted arrow indicates a calcification, with high attenuation on CTA, signal loss on 3D MEDIC and signal loss on T1w. In panel **C**, in green color, the filled arrows on both sides indicate large non-calcified plaque components with signal loss on 3D MEDIC and no elevation of Tw1 signal (**MEDIC + /T1w**). The dotted arrow on the left side indicate a calcification, with high attenuation on CTA, signal loss on 3D MEDIC and signal loss on T1w on the asymptomatic side (this side was not included in final image analysis). In panel **D**, in white color, the arrow indicates a small non-calcified plaque without signal loss on 3D MEDIC and no T1w signal elevation (**MEDIC-/T1w-**)

The results of the image analysis are shown in Table 4 and the corresponding typical patterns are illustrated in Fig. 1.

**Table 4:**
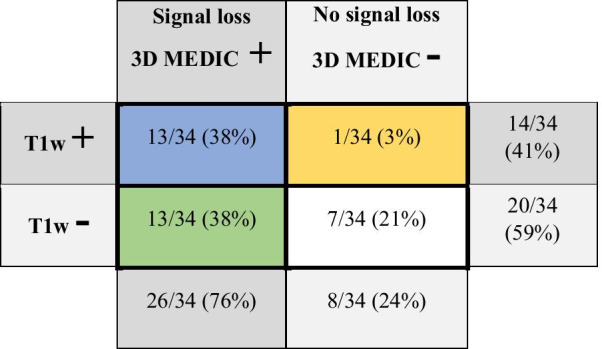
The distribution of signal loss on 3D MEDIC and elevated T1w signal on the T1wBB

Cross table with the distribution of the presence or absence of signal loss on 3D MEDIC and T1w signal elevation in the 34 plaques. The four groups have the same color representation as used in Fig. 1

The column **3D MEDIC + **represents signal loss on 3D MEDIC. The row **T1w + **represents presence of T1w signal elevation on T1wBB.

The blue box in Table 4 shows the proportion of plaques n (%) with signal loss on 3D MEDIC and elevated T1w signal on T1wBB. The green box indicates the proportion of plaques with signal loss without elevated T1w signal. The white box indicates the proportion of plaques without signal loss and no elevation of T1w signal. The Yellow box show the one plaque with elevated T1w signal without signal loss on 3D MEDIC.

The median degree of stenosis, calculated by the common carotid artery method, was 70% on CTA (IQR = 50–88%) as well as on MRI (IQR = 60–88%).

All areas with macro-calcifications on CTA showed corresponding signal loss on 3D MEDIC. The majority of plaques (26/34, 76%) had signal loss within the non-calcified component, suggestive of the presence of hemosiderin, on 3D MEDIC. Less than half of the plaques 14/34 (41%) had an elevated T1wBB signal within the non-calcified component, suggestive of the presence of methemoglobin.

Cohen’s kappa for inter-reader agreement was 0.64 (CI 0.345–0.925) for 3D MEDIC and 0.94 (CI 0.81–1.00) for T1wBB.

## Discussion

This is an explorative observational study to test the feasibility of a spoiled gradient echo based T2* weighted MRI sequence (3D MEDIC) for carotid plaque imaging, and to compare signs suggestive of hemosiderin on 3D MEDIC with signs of methemoglobin on a T1wBB sequence.

The patients were selected based on the presence on non-calcified plaque components on CTA and with recent TIA or stroke. This designed selection bias was intended to promote an enriched prevalence of IPH in the material.

The anatomy of the common carotid was often asymmetric, both in terms of angle but often also not exactly symmetrical in level. Correct coil placement was very important. The closer the coil was to the vessel, the better was SNR and CNR (visually assessed). We always aimed at acquiring high-quality scan of both sides but in situations when the patient had a short neck, the symptomatic side was prioritized and the patient would have to tilt the head slightly to the contralateral side for the surface coil to fit on the symptomatic side. This set some limitations with sometimes suboptimal coil placement on the non-symptomatic side.

During planning of the axial slices of the 3D MEDIC and the T1wBB, the vessel on the symptomatic side, seen on the TOF sequence, was used to set the axial slices perpendicular to the common carotid artery. This could affect the result with a slightly oblique slice plane of the T1wBB images on the non-symptomatic side. This was less of a problem for the 3D MEDIC, which was volumetric, and could be reconstructed in MPR.

The degree of technical feasibility for 3D MEDIC, defined as images acceptable to both neuroradiologist readers, was 34/42 patients (81%). The poorer inter-reader agreement for 3D MEDIC compared to T1wBB may suggest that the image quality was inferior for the 3D MEDIC, even though the images were accepted by both readers. There are other possible explanations for the lower inter-reader agreement, such as lesser experience of 3D MEDIC images.

In atherosclerotic plaques with IPH, different hemoglobin degradation products are known to co-localize [26]. Intracellular methemoglobin, extracellular methemoglobin and hemosiderin affects the signal characteristics on MRI differently [14], and if the degradation products of hemoglobin co-localize, the MRI image can become complex and IPH diagnosis difficult to make. Simpson et al. [27] showed in a study with 37 patients, that the elevated T1w signal was visible for 2 years in the majority of cases.

By applying the 3D MEDIC sequence together with a T1wBB sequence, we show that non-calcified plaques contain tissue that affects susceptibility *without* simultaneous T1-shortening and that this is in fact a relatively common imaging feature present in 13/34 of the plaques in this group of patients. This image features would be suggestive of hemosiderin with little or no methemoglobin. Our study also shows that an equally large group display elevated T1w signal *in combination with* signal loss on 3D MEDIC (Table 4 and Fig. 1), an imaging feature suggestive of hemosiderin and methemoglobin in combination. An alternative explanation for this finding could be the presence of very small amounts of calcium, undetectable on CTA, which have also been demonstrated within atherosclerotic plaques [11]. A recent study by Wang et al. [21] showed co-localization of elevated T1w signal and susceptibility artefacts, similar to our finding, as well as the capability of quantitative susceptibility mapping to discern macro-calcifications from hemorrhage.

In 7/34 of the examined plaques in our study no signal loss on 3D MEDIC or T1w-elevation was seen, which may indicate a more stable plaque composition. Lastly, we found that elevated T1w signal *without* signal loss on 3D MEDIC was an uncommon finding in this group of patients, only seen in one plaque in our study.

There are several limitations of this study. There was no histological or biochemical verification of the imaging findings, which would eventually be desired to support the hypothesis that 3D MEDIC can be used to identify hemosiderin. This was not possible since only a small portion of the included patients were treated surgically. Another limitation is that a SWI sequence was not included in the imaging protocol. This would be desirable to compare the technical feasibility with 3D MEDIC. In our experience successful high-resolution SWI imaging of carotid plaques in 34 out of 42 patients would be challenging with SWI sequences, but this remains to be further studied.

## Conclusion

This study show that high-resolution plaque imaging using 3D MEDIC is feasible in the large majority of patients, and that the properties of the sequences used here theoretically may separately identify the different hemoglobin degradation products methemoglobin and hemosiderin, thereby potentially adding information of IPH and prolonging the period when IPH can be visualized on MRI.

## Data Availability

The data that support the findings of this study are available from the corresponding author, upon reasonable request.

## Abbreviations

CI: Confidence interval
CSF: Cerebrospinal fluid
CTA: Computed tomography angiography
FoV: Field of view
Gd: Gadolinium
HU: Hounsfield unit
ICA: Internal carotid artery
IPH: Intra plaque hemorrhage
IQR: Interquartile range
MEDIC: Multi-Echo Data Image combination
MRI: Magnetic resonance imaging
ms: Millisecond
PACS: Picture Archiving andCommunication System
SPGR: Spoiled gradient echo
SWI: Susceptibility weighted imaging
TE: Time of echo
TIA: Transient ischemic attack
TOF 3D: Time of flight in 3D
TR: Repetition time
TSE2D T1wBB: Turbo spin echo 2D T1 weighted black blood
T2*: T2 star
α°: Flip angle
